# Low femoral antetorsion as a risk factor for bony impingement after bipolar hemiarthroplasty

**DOI:** 10.1186/s13018-015-0248-y

**Published:** 2015-07-07

**Authors:** Takeshi Shoji, Yuji Yasunaga, Takuma Yamasaki, Soutarou Izumi, Susumu Hachisuka, Mitsuo Ochi

**Affiliations:** Department of Orthopaedic Surgery, Graduate School of Biomedical Sciences, Hiroshima University, 1-2-3 Kasumi, Minami-ku, Hiroshima 734-8551 Japan; Department of Orthopaedic Surgery, Hiroshima Prefectural Rehabilitation Center, 295-3, Taguchi, Saijou-town, East Hiroshima, Hiroshima Prefecture Japan

**Keywords:** Bipolar hemiarthroplasty, Three-dimensional motion analysis, Femoral antetorsion, Bony impingement, Dislocation, Impingement pattern

## Abstract

**Introduction:**

Reports of dislocation after bipolar hemiarthroplasty (BHA) abound in literature, and several studies have mentioned the factors that are associated with an increased risk of dislocation. However, there is no report detailing the pattern of impingement in BHA and how femoral antetorsion can affect the range of motion (ROM) after BHA.

**Purpose:**

The purpose of this study was to evaluate the pattern of impingement in BHA and whether femoral antetorsion affects the ROM after BHA using three-dimensional (3D) dynamic motion analysis.

**Methods:**

Using the computed tomography (CT) data of 60 patients (60 hips), including 31 men and 29 women who underwent BHA for the treatment of idiopathic osteonecrosis (ION) of the femoral head, we calculated the antetorsion of the femoral neck, ROM of flexion (Flex), internal rotation (Int-R), and external rotation (Ext-R) using a CT-based 3D simulation software. We evaluated the pattern of impingement and the relationship between femoral antetorsion and ROM in BHA. As for the implant position in the 3D simulation software, the anteversion of the femoral implant was set to be the same as the natural antetorsion of the femoral neck and neck length was set to be the standard neck in all cases.

**Results:**

This study revealed the mechanism of impingement in BHA: (1) bone to bone impingement and (2) implant to bone impingement. We found a significant decrease in the ROM of Flex and Int-R inversely proportional to the femoral antetorsion. In patients with lower femoral antetorsion, the ROM of Flex and Int-R decreased due to bony impingement (the anterior great trochanteric region of the femur impinges on the anteroinferior edge of the anteroinferior iliac spine). Whereas, high anteversion of the femoral implant may decrease the ROM of Ext-R; however, our results also showed that even the lowest ROM of Ext-R with 10° hip extension was over 40°.

**Conclusions:**

We demonstrated that lower femoral antetorsion substantially affects the ROM of Flex and Int-R due to bony impingement. For these patients, there should be consideration given to retaining femoral “anterior offset” in BHA.

## Introduction

Bipolar hemiarthroplasty (BHA) was initially used to treat displaced femoral neck fractures in elderly patients [1], and several series have demonstrated predictable pain relief, better functional outcomes, and fewer reoperations [2, 3]. BHA has been gradually applied to osteonecrosis (ON) of the femoral head [4, 5], and it is sometimes used for treating ON patients in Japan. The prosthesis consists of two articulating surfaces: one between the femoral head and polyethylene liner and one between the metallic shell and acetabulum. In theory, BHA has an additional articulating joint within the head, thereby allowing movement to occur both at the prosthesis acetabular interface and within the prosthesis. In addition, the metallic shell has a large diameter; therefore, BHA was thought to have an advantage in improving stability of the prosthesis and resistance to dislocation. In fact, Parvizi et al. reported that BHA was used successfully in treating recurrent instability after total hip arthroplasty (THA) [6]. However, the published incidence of dislocation after BHA has a wide range from 1 to 15 % [7–9], and when this complication has occurred, the risk of recurrent dislocation was high [10]. Reports of dislocation after BHA abound in literature, and several studies have mentioned the factors that are associated with an increased risk of dislocation such as surgical approach, patient-related factors, and implant malpositioning [1, 4, 11]. However, no reports have detailed the mechanism of dislocation and the optimal setting of a femoral implant in BHA.

Nowadays, preoperative planning is often carried out, and computer simulation analysis is used by several investigators to predict optimal implant settings and to analyze the range of motion (ROM) in THA [12–15]. In this study, we took a subject-specific approach to evaluate the pattern of impingement and the influence of femoral antetorsion on restricting hip ROM after BHA using computed tomography (CT)-based three-dimensional (3D) dynamic motion analysis.

## Patients and methods

In our institute, BHA was performed for specific patients because we think it is a reasonable alternative for the treatment of ON even in a young patient [5, 16]. In this study, we reviewed a total of 60 patients (60 hips), including 31 men and 29 women who underwent BHA for the treatment of idiopathic osteonecrosis (ION) of the femoral head. The mean age at surgery was 48.6 years old (24 ~ 74 years old). The diagnosis of ION was based on the clinical presentation and imaging studies, including plain radiographs and MRI findings [17]. The classification according to the Japanese Investigation Committee of Health and Welfare [18] was stage 3B in all patients, which represented the collapse of the femoral head more than 3 mm. We excluded patients who had undergone previous surgery from the present study. A subset of patients with complete implant data was reviewed for sizing in 3D motion analysis. All patients had a preoperative CT scan of their hip joint, from the anterior superior iliac spine (ASIS) to the knee joint through the distal femoral condyles using a 320-row multi-detector helical CT scanner (Aquilion ONE, Toshiba Medical healthcare, Tochigi, Japan) (detector configuration: 80 × 0.5, beam collimation: 40 mm) with a reconstructed slice width of 1.00 mm and a slice interval of 1.00 mm. The CT data were transferred to the planning module. Ethics approval was granted by the Institutional Review Board of Hiroshima University.

### Three-dimensional motion analysis

A computed tomography-based simulation software (ZedHip Lexi Co., Ltd., Tokyo, Japan) [19] was used to create virtual 3D bone models and to perform virtual simulations of the femoral cut and implant setting, using the preoperative BHA planning mode. This software allows for the generation and separation of independent femoral and acetabular 3D models.

**Fig. 1:**
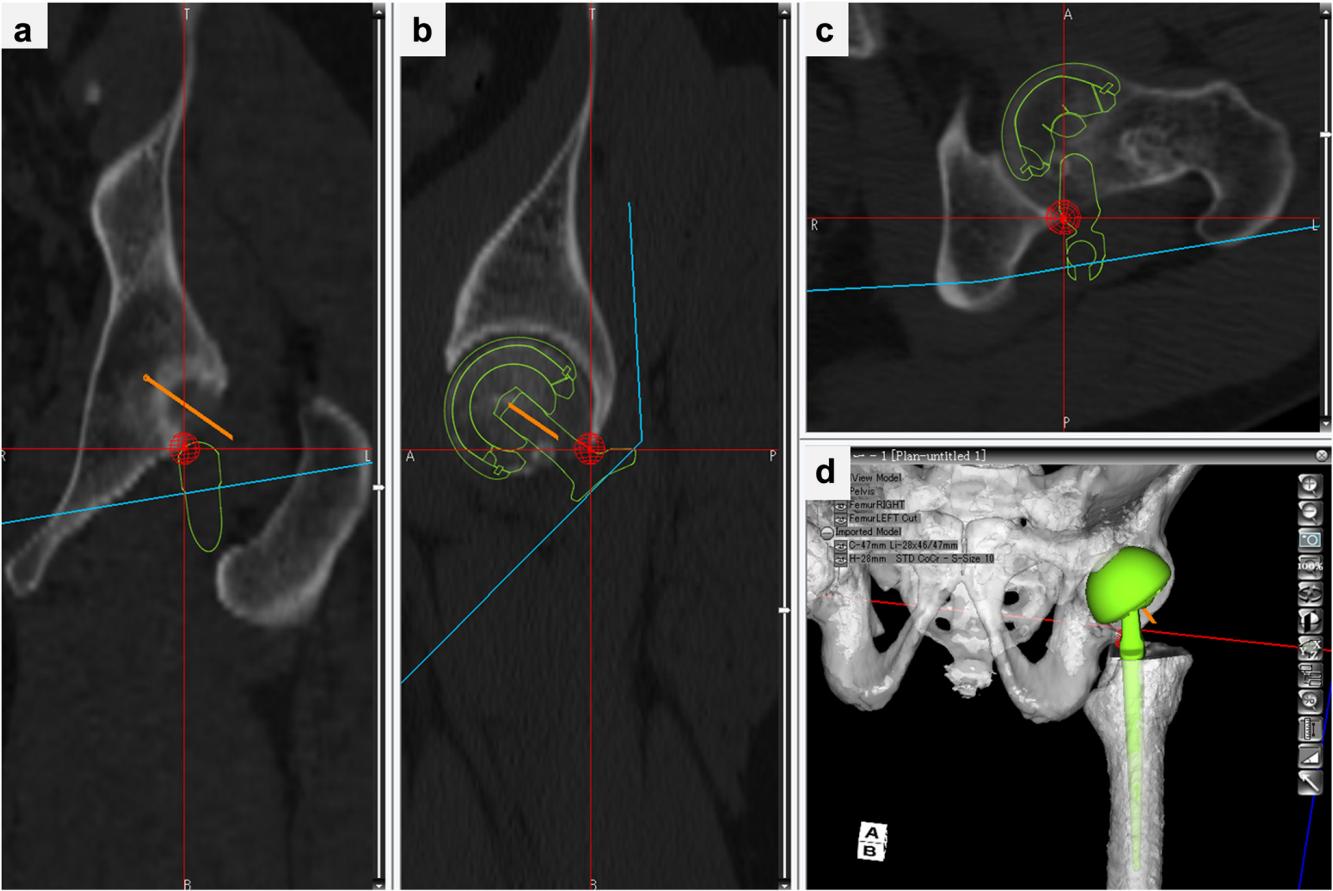
3D simulation of hip ROM and detection of impingement site in BHA. Neck of femoral implant impinges on posterior edge of acetabulum in external rotation with 10° extension. **a** Coronal view. **b** Sagittal view. **c** Axial view. **d** 3D model

Based on a CT scan of the pelvis and femur, the reference points were firstly digitized, then, a 3D reconstruction of the bone model was made semi-automatically. If there was noise, the latter was revised manually. Next, the size of the implants and their 3D orientation relative to the host bones were planned, and implantation was performed in a multi-planar reconstruction (MPR) view. This software enables the simulation and calculation of the ROM until contact occurs between the bones and component. It also visualizes the site of impingement in 3D axial/sagittal/coronal views of MPR images (Fig. 1). The pelvic coordinate system was the functional pelvic plane, and the femoral coordinate system was defined by the center of the femoral head, the knee center, and both femoral condyles. The antetorsion angle of the femoral neck to the transepicondylar axis of the knee was measured as a parameter of the native antetorsion on the axial plane in the simulation.

### Implant setting

The simulated implant was the TAPERLOC® Complete stem with a 28-mm-diameter alumina head, a neck of standard length, and a Ringloc Bipolar shell with a PE insert (Biomet, Warsaw, USA) in all cases. The femoral implant size was chosen to maximize both fit and fill in the femoral metaphysis under the consideration of the implant size used in the operation. The bipolar shell size was also chosen to maximize both fit and fill in the femoral head under the consideration of the shell size used in the operation. As for the position of the implant, the shaft axis of the femoral implant was placed in the center of the original femoral diaphysis, while anteversion was set to be the same as the femoral neck’s anatomical rotation in all cases. The bipolar shell position was determined to be the place at the site of the original femoral head. Any acetabular osteophytes that were attached to the acetabular bony rim were removed.

### Calculation of the ROM and impingement site

The pelvis was fixed in space, while the femur was free to translate in all directions but was constrained to rotate around the center of rotation of the hip. The computer software was capable of detecting both bone to bone and bone to implant impingement, which allowed the maximum ROM to be defined as the degrees of movement before impingement occurred. The location of this impingement on both the femoral and acetabular sides, as well as the position of the femur in space relative to the fixed pelvis, can be also defined in the model. Based on this computerized analysis, the ROM was measured in those directions that are important for dislocation and activity of daily life (ADL): flexion (Flex) with 0° of adduction and internal rotation, internal rotation (Int-R) in 90° of flexion with 0° of adduction, and external rotation (Ext-R) in 10° of extension with 0° of adduction.

### Evaluation design

To evaluate the pattern of impingement and the relationship between femoral antetorsion and ROM, three evaluations were performed in this study:

1. Analysis of the pattern of impingement after BHA.
2. Analysis of the relationship between ROM and femoral antetorsion.
3. We defined the low angle of antetorsion of the femur (≦10°) and the high angle of antetorsion of the femur (≧25°) according to the previous report [20]. We compare the ROM among patients with a low angle of antetorsion of the femur (≦10°) (low-antetorsion group), normal-antetorsion group (10°<, <25°), and a high angle of antetorsion of the femur (≧25°) (high-antetorsion group).

### Statistical analysis

All data were expressed as mean ± standard deviation (SD), and statistical analysis was performed using Stat-View-J version 5.0 software (Hulinks, Tokyo, Japan). The correlations were evaluated using Pearson’s chi-squared test. A *P* value of less than 0.05 was considered statistically significant.

## Results

The mean femoral antetorsion was 12.2° ± 8.3° in men and 23.8° ± 9.7° in women. There was a significant difference between the antetorsion of men and that of women. There was no significant correlation between femoral antetorsion and age or height. However, there was a significant negative correlation between femoral antetorsion and the stem or cup size (Table 1).

**Table 1:** The relationship between femoral antetorsion and each parameter

	*P* value	*r*
Age	0.19	−0.17
Height	0.12	−0.2
Stem size	<0.05	−0.29
Cup size	<0.05	−0.42

### Analysis of the pattern of impingement after BHA

**Fig. 2:**
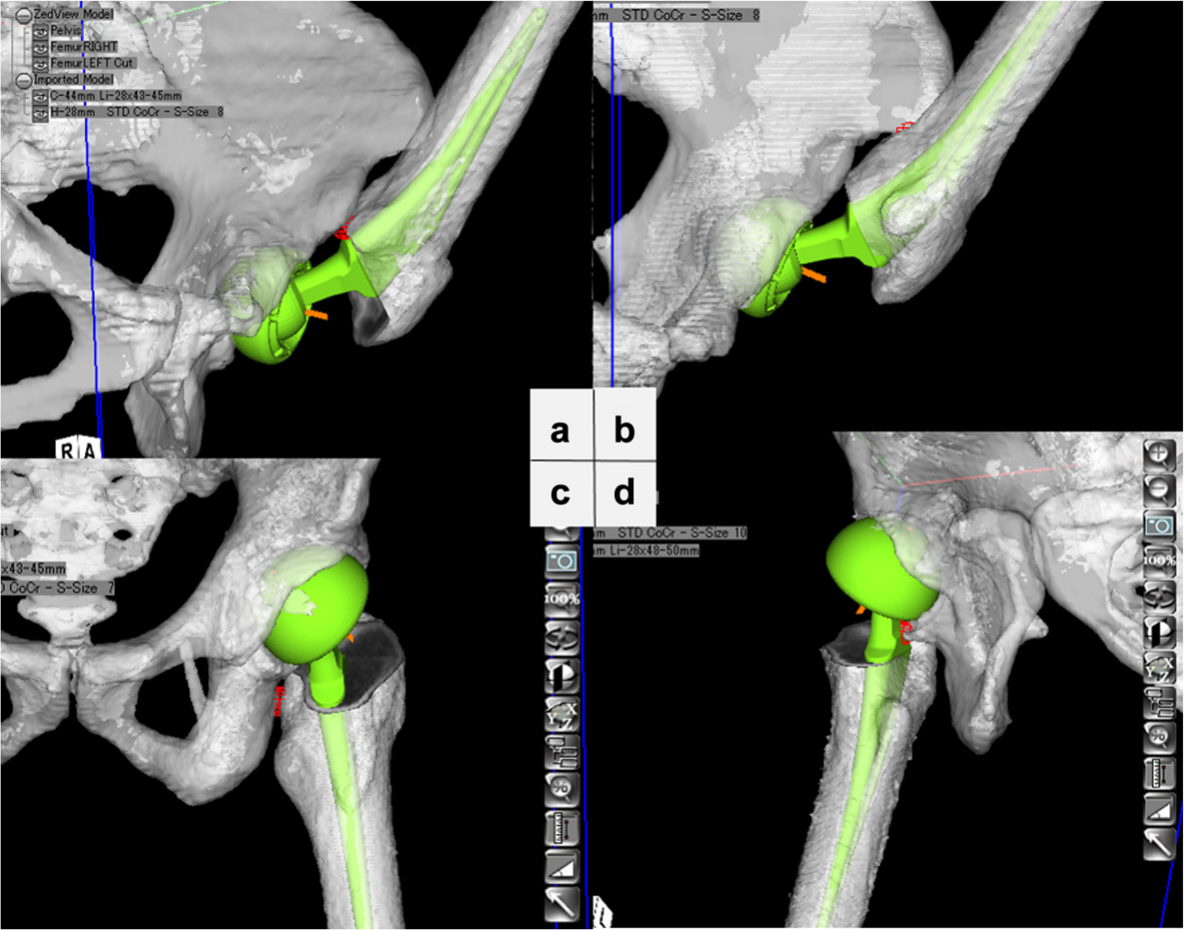
Pattern of impingement after BHA. **a** Bony impingement (femoral neck impinges on the AIIS). **b** Bony impingement (femoral shaft impinges on the ASIS) **c** Bony impingement (posterior aspect of greater trochanter impinges on the ischial bone). **d** Implant-bone impingement (implant neck—posterior part of the acetabulum)

Impingement occurred in two ways: bone to bone impingement and implant to bone impingement. In Flex, bony impingement occurred in all cases. In 51 of 60 cases, the anterior aspect of the greater trochanter or femoral neck at the cutting point impinges on the anteroinferior edge of the anteroinferior iliac spine (AIIS), and in the remaining of 9 cases, the femoral shaft impinges on the ASIS (Fig. 2a, b). In Int-R, the anterior greater trochanteric region of the femur or femoral neck at the cutting point impinges on the anteroinferior edge of the AIIS in all cases (Fig. 2a). In Ext-R, bony impingement—posterior aspect of the greater trochanter impinges on the ischial bone—occurred in 46 cases (Fig. 2c) and implant to bone impingement—posterior aspect of the implant neck impinges on the posterior edge of the acetabulum—occurred in the remaining of 14 cases (Fig. 2d).

### Analysis of the relationship between ROM and femoral antetorsion

**Fig. 3:**
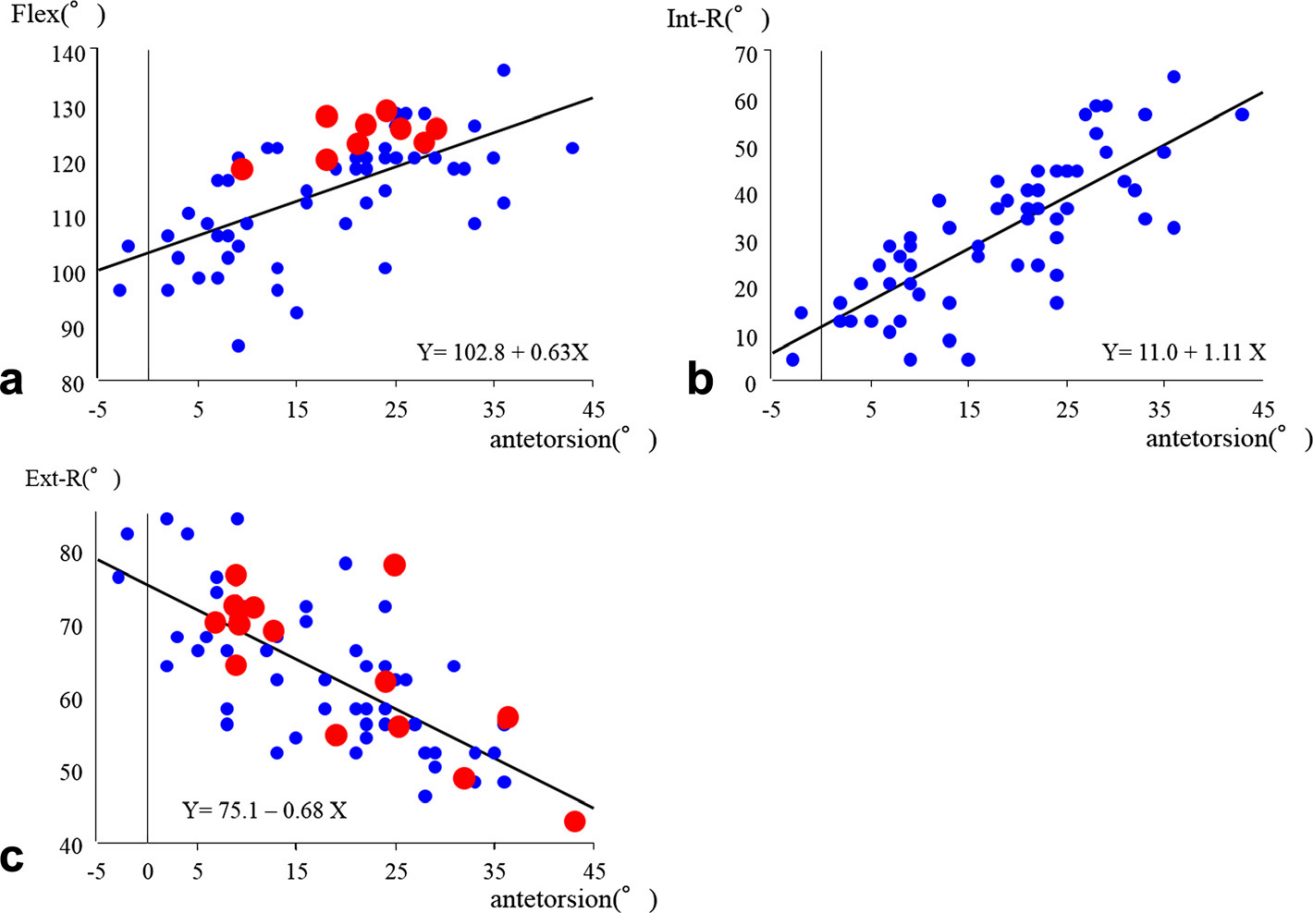
The relationship between ROM and femoral antetorsion. **a** Flex-antetorsion. *Blue dots* indicate bony impingement (greater trochanter on the AIIS) and *red dots* indicate bony impingement (femoral shaft impinges on the ASIS). **b** Int-R-antetorsion. *Blue dots* indicate bony impingement (greater trochanter or femoral neck on the AIIS). **c** Ext-R-antetorsion. *Blue dots* indicate bony impingement and *red dots* indicate implant neck to bone impingement

The mean ROM in simulation was 114.1° ± 10.1° in Flex, 31.1° ± 15.1° in Int-R, and 62.8° ± 10.3° in Ext-R. There were positive correlations between the femoral antetorsion and the ROM of Flex (*R*^2^, 0.39; *P* < 0.0001), and an even stronger correlation between the femoral antetorsion and the ROM of Int-R (*R*^2^, 0.62; *P* < 0.0001), respectively (Fig. 3a, b). Whereas, there was negative correlation between the femoral antetorsion and the ROM of Ext-R (*R*^2^, 0.50; *P* < 0.0001) (Fig. 3c). Some cases produced results of a maximum of 10° in Int-R, especially in cases with excessively low femoral antetorsion (Fig. 3b). When the site of impingement was plotted to the previous graph (Flex-antetorsion and Ext-R-antetorsion), there was no tendency in either of the two groups (Fig. 3a, c).

### Comparison of the ROM among three groups

**Fig. 4:**
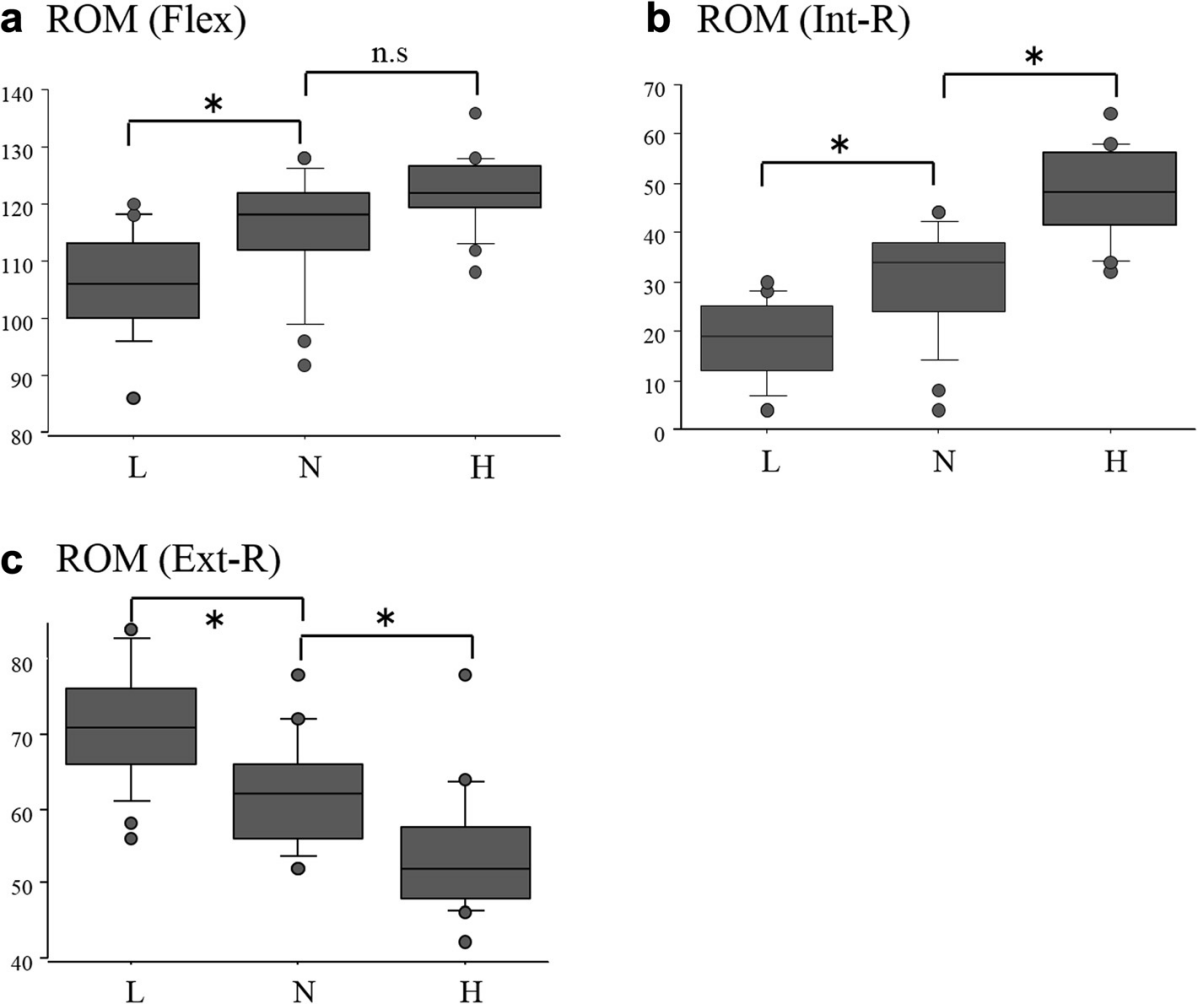
ROM of each group. *L* indicates the low-antetorsion group, *N* indicates the normal-antetorsion group, and *H* indicates the high-antetorsion group. **a** The ROM of Flex. **b** The ROM of Int-R. **c** The ROM of Ext-R

The low-antetorsion group included 20 hips (16 men and 4 women), the normal-antetorsion group included 23 hips (14 men and 9 women), and the high-antetorsion group included 17 hips (1 man and 16 women). The mean antetorsion was 5.9° ± 3.7° in the low-antetorsion group, 19.3° ± 4.1° in the normal-antetorsion group, and 30.6° ± 5.0° in the high-antetorsion group. In the normal-antetorsion group, the mean ROM was 115.3° ± 10.0° in Flex, 30.1° ± 11.1° in Int-R, and 61.6° ± 7.1° in Ext-R. In the high-antetorsion group, the mean ROM was 122.2° ± 6.6° in Flex, 47.8° ± 9.4° in Int-R, and 54.4° ± 8.5° in Ext-R, while it was 105.9° ± 8.8° in Flex, 18.0° ± 7.9° in Int-R, and 64.5° ± 8.0° in Ext-R in the low-antetorsion group. There were significant differences among the three groups in terms of the ROM of Int-R and Ext-R (Fig. 4a–c). Whereas, in the ROM of Flex, there were significant differences between the low-antetorsion group and the normal-antetorsion group and between the low-antetorsion group and the high-antetorsion group. As for the ROM of Flex and Int-R, the ROM was highest in the high-antetorsion group. On the other hand, the ROM of Ext-R was highest in the low-antetorsion group.

## Discussion

Since its development in the early 1970s, BHA has gained in popularity for the treatment of ON of the femoral head and good results have been reported [16, 21]. However, there are several complications in BHA. Dislocation, infection, and periprosthetic fracture are the main reasons for failure after BHA surgery. Each complication appears to have a particular risk profile. With regard to dislocation, the dislocation rate is reportedly increased by various factors such as age, medical condition, mental disorder, and surgical approach [1, 4, 11, 22, 23]. In addition, anatomical factors such as acetabular measurements indicative of hip dysplasia were also reported to contribute to dislocation [24].

Impingement is often the main etiology of post-surgery instability. Dislocation can occur subsequent to impingement between the components, the acetabulum, and the proximal femur. Multifold models have been developed to determine the optimal implant setting for maximizing ROM and minimizing the risk of impingement in THA; however, there has been minimal reporting on the mechanism of dislocation and the optimal setting of a femoral implant in BHA.

Nowadays, preoperative planning can be executed with high accuracy in THA, and the optimal implant orientation and impingement have been evaluated by many investigators using computerized simulation analysis [12–15]. This form of analysis offers a template of information regarding the location of the impingement region which provides feedback on the anticipated improvement in motion, in vivo. Our computer model has been used to evaluate the pattern of impingement and the resultant ROM after BHA.

In our study, bone to bone impingement or implant to bone impingement initially occurred in Flex, Int-R, and Ext-R. In THA, Bartz et al. noted three different mechanisms of dislocation: (1) prosthetic impingement, (2) bony impingement, and (3) spontaneous dislocation [25]. In BHA, logically, implant impingement may not contribute to dislocation; therefore, we can classify the mechanism of impingement in BHA from this study as follows: (1) bone to bone impingement, (2) implant to bone impingement, and (3) spontaneous dislocation. This suggests that the factors of dislocation are prescribed by the implant shape, setting position of stem, head size, and hip bone morphology. Our results showed that the lower the antetorsion of the femoral implant is, the more the ROM of Flex and Int-R decreases. Whereas, high antetorsion of the femoral implant may decrease the ROM of Ext-R. However, our results also showed that even the lowest ROM of Ext-R with 10° hip extension was over 40° in patient with 43° of antetorsion. Miki et al. reported that the anatomical hip ROM in patients after THA was up to 36° in external rotation with 0° hip extension [26], which means that our Ext-R ROM results were sufficient to enable everyday activities. These results indicate that lower femoral antetorsion has a risk for restricting hip ROM in Flex and Int-R due to bony impingement. However, high anteversion (<40°) may not lead to impingement in BHA. Furthermore, the result that femoral antetorsion correlates negatively with the male gender and implant size also indicates that men with larger bone morphology have a risk for anterior impingement.

We define “anterior offset” as the distance between the line on the anterior aspect of the proximal femur and the center of the head, and it is important to retain this “anterior offset” in order to avoid bony impingement and to improve ROM of Flex and Int-R especially in patients with lower femoral antetorsion. Our results suggest that elongation of the stem offset and/or the use of a femoral implant with setting appropriate anteversion may increase the hip ROM after BHA. If bony impingement is observed as a restricting factor in these conditions, resection of the bony impingement site (the anterior aspect of the femoral neck and greater trochanter or anteroinferior aspect of the AIIS) may reduce the incidence of posterior dislocation by allowing an increase of ROM in Flex and Int-R until bony impingement. This would be of serious concern for patients with a low femoral antetorsion, and these factors can and must be taken into consideration preoperatively during the planning of BHA, especially for patients with larger bone morphology.

There were several limitations in our study. Firstly, the influence of the surrounding soft tissue was not taken into account, which may have affected the actual hip ROM. Secondly, we only analyzed the ROM until impingement. Hip dislocation involves levering the head out of the outer cup after impingement, so a larger outer head in BHA may have some advantage of reducing dislocation by way of providing a jumping distance. Thirdly, the number of patients with high antetorsion was not large enough to enable assessment of patients with excessive high antetorsion. Fourth, pelvic inclination was not taken into consideration in our study. In elderly patients, we often see the posterior tilt of the acetabulum, so an excessive antetorsion of the femur may run the risk of posterior impingement.

## Conclusion

In summary, we demonstrated that the antetorsion of the femoral implant substantially affects ROM of Flex and Int-R especially in patients with a low antetorsion of the femur. When using cementless bipolar or unipolar hip arthroplasty to patients with low femoral antetorsion, even if the patient is with femoral neck fracture, the orthopedic surgeon should consider the possibility of impingement. Certain precautions must be followed during the operation to retain the femoral “anterior offset” by using a femoral implant with increased anteversion, cemented implant, elongation of the stem offset, and/or the resection of the bony impingement site in BHA.

## References

[1] Pajarinen J, Savolainen V, Tulikoura I, Lindahl J, Hirvensalo E (2003). Factors predisposing to dislocation of the Thompson hemiarthroplasty. Acta Orthop.

[2] Frihagen F, Nordsletten L, Madsen JE (2007). Hemiarthroplasty or internal fixation for intracapsular displaced femoral neck fractures: randomised controlled trial. BMJ.

[3] Parker MJ, Gurusamy K (2006). Internal fixation versus arthroplasty for intracapsular proximal femoral fractures in adults. Cochrane Database Syst Rev.

[4] Enocson A, Hedbeck CJ, Törnkvist H, Tidermark J, Lapidus LJ (2012). Unipolar versus bipolar Exeter hip hemiarthroplasty: a prospective cohort study on 830 consecutive hips in patients with femoral neck fractures. Int Orthop.

[5] Moriya M, Uchiyama K, Takahira N, Fukushima K, Yamamoto T, Hoshi K (2012). Evaluation of bipolar hemiarthroplasty for the treatment of steroid-induced osteonecrosis of the femoral head. Int Orthop.

[6] Parvizi J, Morrey BF (2001). Bipolar hip arthroplasty as a salvage treatment for instability of the hip. J Bone Joint Surg Am.

[7] Varley J, Parker MJ (2004). Stability of hip hemiarthroplasties. Int Orthop.

[8] Madanat R, Mäkinen T, Ovaska M, Soiva M, Vahlberg T, Haapala J (2012). Dislocation of hip hemiarthroplasty following posterolateral surgical approach: a nested case-control study. Int Orthop.

[9] Rogmark C, Fenstad A, Leonardsson O, Engesæter L, Kärrholm J, Furnes O (2014). Posterior approach and uncemented stems increases the risk of reoperation after hemiarthroplasties in elderly hip fracture patients. Acta Orthop.

[10] Barnes C, Berry D, Sledge C (1995). Dislocation after bipolar hemiarthroplasty of the hip. J Arthroplasty.

[11] Enocson A, Tidermark J, Törnkvist H, Lapidus LJ (2008). Dislocation of hemiarthroplasty after femoral neck fracture: better outcome after the anterolateral approach in a prospective cohort study on 739 consecutive hips. Acta Orthop.

[12] Widmer KH, Zurfluh B (2004). Compliant positioning of total hip components for optimal range of motion. J Orthop Res.

[13] Kessler O, Patil S, Stefan W, Mayr E, Colwell CW, Darryl D (2008). Bony impingement affects range of motion after total hip arthroplasty: a subject-specific approach. J Orthop Res.

[14] Rousseau MA, Lazennec JY, Boyer P, Mora N, Gorin M, Catonné Y (2009). Optimization of total hip arthroplasty implantation: is the anterior pelvic plane concept valid?. J Arthroplasty.

[15] Incavo SJ, Thompson MT, Gold JE, Patel RV, Icenogle KD, Noble PC (2011). Which procedure better restores intact hip range of motion: total hip arthroplasty or resurfacing? A combined cadaveric and computer simulation study. J Arthroplasty.

[16] van Egmond PW, Taminiau AH, van der Heide HJ (2013). Hemiarthroplasties in young patients with osteonecrosis or a tumour of the proximal femur; an observational cohort study. BMC Musculoskelet Disord.

[17] Sugano N, Kubo T, Takaoka K, Ohzono K, Hotokebuchi T, Matsumoto T (1999). Diagnostic criteria for non-traumatic osteonecrosis of the femoral head: a multicenter study. J Bone Joint Surg (Br).

[18] Sugano N, Atsumi T, Ohzono K, Kubo T, Hotokebuchi T, Takaoka K (2002). The 2001 revised criteria for diagnosis, classification, and staging of idiopathic osteonecrosis of the femoral head. J Orthop Sci.

[19] Shoji T, Yasunaga Y, Yamasaki T, Mori R, Hamanishi M, Ochi M (2013). Bony impingement depends on the bone morphology of the hip after total hip arthroplasty. Int Orthop.

[20] Fabry G, MacEwen GD, Shands AR (1973). Torsion of the femur. A follow-up study in normal and abnormal conditions. J Bone Joint Surg Am.

[21] Hwang K, Kim Y, Kim Y, Choi I (2012). Is bipolar hemiarthroplasty a reliable option for Ficat stage III osteonecrosis of the femoral head? 15- to 24-year follow-up study. Arch Orthop Trauma Surg.

[22] Lung HR (1971). The role of prosthetic replacement of the head of the femur as primary treatment for subcapital fractures. Injury.

[23] Chana R, Mansouri R, Jack C, Edwards MR, Singh R, Keller C, et al. The suitability of an uncemented hydroxyapatite coated (HAC) hip hemiarthroplasty stem for intra-capsular femoral neck fractures in osteoporotic elderly patients: the Metaphyseal-Diaphyseal Index, a solution to preventing intra-operative periprosthetic fracture. J Orthop Surg Res. 2011; doi:10.1186/1749-799X-6-59. 10.1186/1749-799X-6-59 PMC3231806 22099169

[24] Christopher C, Sethi N, Hatahet M, Clifford L, Morandi M, Vaidya R (2009). Hip dislocation after modular unipolar hemiarthroplasty. J Arthroplasty.

[25] Bartz RL, Nobel PC, Kadakia NR, Tullos HS (2000). The effect of femoral component head size on posterior dislocation of the artificial hip joint. J Bone Joint Surg Am.

[26] Miki H, Yamanashi W, Nishii T, Sato Y, Yoshikawa H, Sugano N (2007). Anatomic hip range of motion after implantation during total hip arthroplasty as measured by a navigation system. J Arthroplasty.

